# Nondipping in Parkinson's Disease

**DOI:** 10.4061/2011/897586

**Published:** 2011-09-18

**Authors:** Sita Sommer, Billur Aral-Becher, Wolfgang Jost

**Affiliations:** ^1^Department of Neurology, Universitätsmedizin Mainz, Langenbeckstraße 1, 55131 Mainz, Germany; ^2^Department of Neurology, Deutsche Klinik für Diagnostik, Aukammallee 33, 65191 Wiesbaden, Germany

## Abstract

*Objective*. The aim of this study was to identify patients with Parkinson's disease who showed loss or decrease of nocturnal blood pressure fall (nondipper patients) as a marker of autonomic dysfunction. Presence or absence of orthostatic hypotension was considered to investigate whether alterations in circadian blood pressure pattern are associated with posture-related dysregulation of blood pressure. *Methods*. 40 patients with Parkinson's disease underwent 24-hour blood pressure monitoring. 21 patients were diagnosed with arterial hypertension and received anti-hypertensive drugs. Nondipper patients were defined as having nocturnal decrease of mean systolic and diastolic blood pressure less than 10%. Presence or absence of orthostatic hypotension was determined by Schellong's test. *Results*. We identified 35 nondipper patients (88%). Nondipping was detected in 20 patients with orthostatic hypotension (95%) and in 15 patients without orthostatic hypotension (79%). 18 patients with hypertensive and 22 patients with normal blood pressure values were detected. *Conclusions*. In conclusion 24-hour blood pressure monitoring showed a high prevalence of nondipping in 40 patients with Parkinson's disease with and without orthostatic hypotension independent of coexisting arterial hypertension and antihypertensive treatment. 24-hour blood pressure monitoring may be useful to identify non-dipping as a marker of autonomic dysfunction in patients with Parkinson's disease.

## 1. Introduction

Parkinson's disease is a multisystem degeneration [1]. Beside motor symptoms, and psychiatric symptoms autonomic dysfunction is a common finding in Parkinson's disease [2]. Neuropathological studies showed the presence of Lewy bodies in central and peripheral structures involved in autonomic regulation [3–6].

Involvement of the autonomic nervous system (ANS) occurs in the early stages of the disease [2]. 

Cardiovascular dysautonomia, especially orthostatic hypotension, is frequently reported in Parkinson's disease [7].

Involvement of peripheral components of the ANS can be demonstrated by MIBG scintigraphy.

MIBG scintigraphy shows reduced cardiac uptake of MIBG (meta-[123I]iodobenzylguanidine) representing loss of postganglionic myocardial sympathetic nerve fibers in patients with Parkinson's disease and autonomic failure [8]. However these findings are also seen in the early stages of the disease independent of symptoms of cardiovascular dysautonomia [9].

Further effects of autonomic cardiovascular dysfunction are demonstrated in earlier studies such as baroreceptor reflex dysfunction, alterations in circadian blood pressure regulation, and supine hypertension [10–15]. Findings of earlier reports on ambulatory blood pressure monitoring in patients with Parkinson's disease show a varying number of patients with loss of nocturnal blood pressure fall [13–16]. 

Ambulatory blood pressure monitoring is frequently used in the diagnosis and therapy monitoring of arterial hypertension and allows an evaluation of the circadian blood pressure evaluation [17, 18].

The aim of this study was to identify patients with Parkinson's disease who showed loss or decrease of nocturnal blood pressure fall (non dipper patients) as a potential marker of autonomic dysfunction.

Presence or absence of orthostatic hypotension was considered to investigate whether alterations in circadian blood pressure pattern are associated with posture-related dysregulation of blood pressure.

## 2. Methods

40 patients with Parkinson's disease underwent ambulatory 24-hour blood pressure monitoring. Parkinson's Disease was diagnosed according to the criteria of the United Kingdom Parkinson's disease Society Brain Bank [19]. None had atypical features on neurological examination (e.g., cerebellar, pyramidal) excluding diagnosis other than idiopathic Parkinson's disease (e.g., multiple system atrophy). 

20 of the patients were male, 20 were female; they were aged 41 to 82 years (mean age 69,9 years); disease duration (Parkinson's disease) was 1 to 152 months (mean disease duration 49 months) (Table 1). 21 patients (53%) were diagnosed with arterial hypertension and received antihypertensive drugs. 

Long-term blood pressure measurements were performed with “Mobilograph,” an oscillometric recorder (I.E.M. GmbH, Stolberg, Germany). Patients were asked to maintain their normal physical activity. Nondipper patients were defined as having nocturnal decrease of mean systolic and diastolic blood pressure less than 10%. Presence or absence of orthostatic hypotension was determined by Schellong's test (after resting in the supine position for 5 to 10 minutes the patient's blood pressure and heart rate were measured minute by minute for 3 minutes in the supine position and for a total of 8 minutes in the upright position after standing up rapidly); orthostatic hypotension was defined as a minimum decrease in systolic and/or diastolic blood pressure of 20 mmHg or 10 mmHg, respectively, on standing at the third minute [20]. Supine hypertension was defined as systolic and diastolic blood pressure above 140/90 mmHg in the supine position [21].

### 2.1. Statistical Analysis

Results are presented as mean values (± standard deviation (SD)). A correlation analysis was performed between the presence of loss of nocturnal blood pressure fall and disease duration, age and presence of arterial hypertension/use of antihypertensive medication.

## 3. Results

Ambulatory blood pressure was recorded without interruption in all patients with a mean measurement period of 23 hours (± 1,8 hours). The average number of measurements obtained during the 24-hour recording was 88% (± 11). 

Results of the mean diurnal arterial pressure showed 18 patients with hypertensive blood pressure values and 22 patients with normal blood pressure. Beside the 21 patients who were already diagnosed with arterial hypertension 6 patients showed hypertensive blood pressure values in ambulatory blood pressure recording; thus a total of 27 patients (68%) had arterial hypertension (Table 2).

We identified 35 non dipper patients (88%) among 40 examined patients with Parkinson's disease; 25 patients (63%) showed nocturnal increase of blood pressure (Table 2). Correlation analysis found no correlation between presence of loss of nocturnal blood pressure fall (non dipper) and age, disease duration, and presence of arterial hypertension or antihypertensive treatment. 

**Figure 1:**
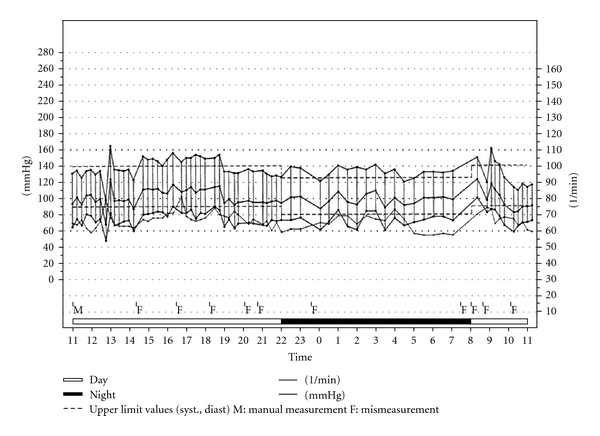
Example of a 24-hour ambulatory blood pressure recording in a patient with Parkinson's disease.

Figure 1 gives an example of a 24-hour blood pressure monitoring in a patient with Parkinson's disease showing loss of nocturnal blood pressure fall.

Autonomic test results showed 21 patients with orthostatic hypotension. Loss of nocturnal blood pressure fall was detected in 20 patients with orthostatic hypotension (95%); this was also detected in 15 patients without orthostatic hypotension (79%). We found 11 patients with supine hypertension who were non dipper (100%) and 24 patients without supine hypertension who were non dipper (83%) (Table 3).

## 4. Discussion

We investigated ambulatory blood pressure monitorings in patients with Parkinson's disease to evaluate the circadian blood pressure profile, especially the presence or absence of loss of nocturnal blood pressure fall (non dipper). Results of earlier studies show a variable percentage of non dipper patients (Table 4), mainly observed in patients with orthostatic hypotension [13–16]. Healthy controls were not required because blood pressure dipping is well known in healthy subjects [22]. 

Our results show that most of our examined patients with Parkinson's disease were non dipper (88%); this was even found in 79% of the patients without orthostatic hypotension. In conclusion, ambulatory blood pressure monitoring could be useful to detect autonomic dysfunction even in the early stages of Parkinson's disease when orthostatic hypotension is not present. 

The alterations in the circadian blood pressure profile may be caused by impairment of the autonomic regulatory mechanisms, possibly through postural dysregulation of blood pressure as mentioned in earlier reports [14]. This dysregulation may produce supine hypertension and nondipping particularly during the night when a supine position is adopted. Furthermore a denervation supersensitivity of the vascular *α*-adrenergic receptors due to sympathetic denervation in patients with Parkinson's disease may contribute to supine hypertension during the night due to exaggerated vasoconstriction [14, 23, 24]. 

Presence or absence of arterial hypertension was additionally investigated in our patients (anamnestic, results of performed blood pressure recordings) to evaluate a possible association with loss of nocturnal blood pressure fall; there was no correlation between the presence of non-dipping and the presence of arterial hypertension or use of antihypertensive medication. 27 patients were additionally diagnosed with arterial hypertension or had hypertensive blood pressure values in ambulatory blood pressure recording (68%). 

24-hour blood pressure monitoring should be performed in patients with Parkinson's disease and coexisting arterial hypertension, who are treated with antihypertensive medication. 

For prevention of an increased prevalence of end-organ damage we propose treatment of nocturnal hypertension in patients with Parkinson's disease. Treatment with antihypertensive drugs in patients with orthostatic hypotension may be complicated by worsening of the postural blood pressure fall in the upright position in these patients and thus may cause syncopes or cerebral ischemia. Consequently, vasoactive drugs should be used carefully in patients with Parkinson's disease and autonomic dysfunction. Alternatively sleeping in a 12° head-up tilt position may be helpful [25]. 

Concerning an increased cardio—and cerebrovascular risk in patients with nondipping, Cornélissen et al. showed that—compared with dipping—abnormal chronobiological end points such as reduced circadian standard deviation of heart rate or elevated pulse pressure are more useful to discriminate between patients who are at a higher risk and who are not [26]. However, to investigate the cardio- and cerebrovascular risk in patients with Parkinson's disease was not aimed in our study. 

In conclusion 24-hour ambulatory blood pressure monitoring showed a high prevalence of non-dipping in 40 patients with Parkinson's disease with and without orthostatic hypotension independent of the coexisting arterial hypertension, and antihypertensive treatment. Our data strengthens the role of ambulatory blood pressure recording for detection of non-dipping not only in patients with orthostatic hypotension, but also in patients without orthostatic hypotension. Beside other autonomic tests (Schellong's test, head-up tilt test) 24-hour blood pressure monitoring may be helpful to support the detection of autonomic dysfunction in patients with Parkinson's disease even in the early stages of the disease.

## Figures and Tables

**Table 1:** Characteristics of the population studied.

Variable	Patients with PD *n* = 40
Age, y	69,9 (8,6)
Sex, M/F	20/20
Disease duration, months	49 (42)

PD: Parkinson's disease; mean values (SD).

**Table 2:** Results of 24 h ambulatory blood pressure monitoring.

Variable	Patients with PD *n* = 40
Cumulative measurement period, hours	23 (1,8)

Mean systolic BP, mmHg	130,3 (9)
Mean diastolic BP, mmHg	77,6 (6,7)

Day (8:00–22:00)	

Mean systolic BP, mmHg	131,4 (10,2)
Mean diastolic BP, mmHg	78,7 (7,3)
Arterial Hypertension: BD systol. > 140/90 mmHg, %	45

Night (22:00–8:00)	

Mean systolic BP, mmHg	129,6 (14,5)
Mean diastolic BP, mmHg	75,4 (9,2)
Pathologic mean nocturnal BP: BP ≥ 125/80 mmHg, %	63

Nocturnal BP	

Non dipper patients, %	88 (*n* = 35)
Nocturnal increase of BP, %	43 (*n* = 17)
Mean systolic decrease of BP at night, %	0,9 (12,9)
Mean diastolic decrease of BP at night, %	3,9 (12,2)

PD: Parkinson's disease; BP: blood pressure; mean values (SD).

**Table 3:** Autonomic test results (Schellong test).

Variable	Patients with PD *n* = 40
Orthostatic hypotension, %	53 (*n* = 21)
OH present + non dipper, %	95 (*n* = 20)
OH absent + non dipper, %	79 (*n* = 15)
Supine hypertension, %	28 (*n* = 11)
SH present + non dipper, %	100 (*n* = 11)
SH absent + non dipper, %	83 (*n* = 24)

PD: Parkinson's disease, OH: orthostatic hypotension, SH: supine hypertension.

**Table 4:** Ambulatory blood pressure monitoring in patients with Parkinson's disease.

Reference	Number of patients	Nondipper (%)
[14]	13	92,3
[15]	24	82
[16]	23	48
[22]	38	63
